# Folate functionalized pH-sensitive photothermal therapy traceable hollow mesoporous silica nanoparticles as a targeted drug carrier to improve the antitumor effect of doxorubicin in the hepatoma cell line SMMC-7721

**DOI:** 10.1080/10717544.2020.1718801

**Published:** 2020-02-03

**Authors:** Yue Cao, Chao Wu, Ying Liu, Lili Hu, Wenjing Shang, Zhanshan Gao, Nan Xia

**Affiliations:** Pharmacy School, Jinzhou Medical University, Jinzhou, Liaoning, China

**Keywords:** Doxorubicin, folic acid-functionalized, pH-sensitive, photothermal therapy, hollow mesoporous silica nanoparticles

## Abstract

In this paper, we prepared doxorubicin-loaded folic acid-functionalized pH-sensitive photothermal therapy (PTT) traceable hollow mesoporous silica nanoparticles (DOX-HPCF) as a drug carrier for liver cancer treatment. According to TEM characterization, hollow mesoporous silica nanoparticles (HMSN) are monodispersed spherical particles with hollow structure. *In vitro* drug release experiments showed that HPCF exhibited pH-sensitive release. Cell uptake experiments showed that HPCF was successfully absorbed by SMMC-7721 cells. In addition, DOX-HPCF significantly inhibited the proliferation of SMMC-7721 cells, and the near-infrared (NIR) light group showed a more obvious inhibitory effect. *In vivo* anti-tumor experiments showed that DOX-HPCF-assisted PTT inhibited tumor growth significantly. Therefore, HPCF is a promising photothermotherapy carrier for the treatment of liver cancer.

## 1. Introduction

Liver cancer is one of the most common cancers in the world and has become the third leading cause of death after lung cancer and stomach cancer (El-Serag & Lenhard Rudolph, 2007). Since more than 80% of deaths occur in developing countries, liver cancer has become a major public health problem in these parts of the world (Chen & Zhang, 2011). Up to now, the main clinical treatment for liver cancer is chemotherapy in addition to surgery. However, most anticancer drugs have high toxicity and low specificity, leading to systemic toxicity and serious side effects (Zhu et al., 2016). To overcome these obstacles, collaborative therapy is considered to improve the therapeutic effect (Fan et al., 2017). In this regard, a variety of collaborative nanoplatforms have been proposed, such as chemical photothermal therapy (PTT) (Lu et al., 2017; Jin et al., 2018), chemical photodynamic therapy (PDT) (Dong et al., 2016; Liu et al., 2017), chemotherapeutic immunotherapy (Zheng et al., 2016), PTT/PDT (Chen et al., 2016a; Liu et al., 2018), etc. Among them, PTT combined with chemotherapy has attracted increasing attention due to its anticancer effect and extremely low damage to normal tissues.

To date, different types of nanomaterials (organic and inorganic) have been studied for cancer co-chemotherapy-PTT, including synthetic polymers (Cai et al., 2017), carbon-based nanostructures (Chen et al., 2016b), mesoporous silica (Mekaru et al., 2015), and nanoscale metallic organic skeletons (Yang et al., 2017). Mesoporous silica nanoparticles (MSNs) have a high surface area, large pore volume, excellent biocompatibility, and easy surface functionalization, etc. (Wu et al., 2013). Compared with MSNs, MSNs with a hollow structure have a larger pore volume and higher drug loading efficiency (Chen et al., 2014).

For cancer chemotherapy-PTT therapy, an ideal photothermal converter is necessary. A range of near-infrared (NIR) light-absorbing inorganic nanomaterials has been widely studied, including gold nanostructures (Luo et al., 2016), carbon nanotubes and graphene oxide (Chen et al., 2015), black phosphorus (Tao et al., 2017), copper sulfide nanoparticles (Liang et al., 2017), and organic nanoparticles such as NIR dyes and polydopamine (PDA) (Zhong et al., 2015; Sheng et al., 2018). As a polymer material, PDA has good biocompatibility, biodegradability and strong NIR absorption, etc. (Shao et al., 2017). Dopamine is present in the adhesive proteins secreted by mussels and usually self-polymerizes to form PDA under alkaline conditions (Zhao et al., 2015). Any ligand molecule containing a nucleophilic functional group such as an amine or a thiol can be fixed to the PDA layer by the Michael addition or the Schiff base reaction (Lee et al., 2007, 2009). Therefore, PDA is a very suitable material for PTT.

With the development of cancer diagnosis and treatment technologies, quantum dots have been widely used as biomarkers. Commonly used quantum dots include CdTe quantum dots (Shi et al., 2014), CdSe quantum dots (Li et al., 2019), and carbon quantum dots (CQDs) (Kim et al., 2013), etc. Among them, CQDs have good water solubility, excellent biocompatibility, and light stability. At the same time, these quantum dots have great potential in the field of bioimaging (Zhou et al., 2013; Hu et al., 2014), so they are very suitable as a tracer material. To further improve the targeting effect of nanoparticles, targeted ligands such as carrier modifiers have been widely investigated. Common target ligands include folic acid (FA), chitosan, ferritin (Federici et al., 2016; Fracasso et al., 2016), iodinated hyaluronic acid, etc. Among them, FA has high affinity with folate receptors (FRs). Since FRs are overexpressed in liver cancer cells (Elnakat & Ratnam, 2006; Maeng et al., 2010), FA is an ideal target for targeting liver cancer.

In this paper, we intended to prepare DOX-HPCF as a drug carrier for liver cancer treatment. An *in vitro* dissolution experiment was conducted to explore the release of DOX. Through *in vitro* cell experiments and *in vivo* tumor-bearing experiments, we examined whether HPCF-assisted PTT can more significantly improve the anti-tumor effect of DOX.

## 2. Materials and methods

### 2.1. Materials

Doxorubicin (DOX, Mw = 543.52, purity ≥ 98%) was provided by Hefei Bomei Biological Technology Co., Ltd. (Hefei, China). [2-(Acryloyoxyethyl)ethyl]trimethylammonium chloride (DAC, 80 wt%) was purchased from Jinan Wanduoxin Chemical Co., Ltd. (Jinan, China). Cetyl trimethyl ammonium bromide (CTAB) was obtained from Tianjin Guangfu Fine Chemical Co., Ltd. (Tianjin, China). 2,2′-Azodiazine(2-methylpropiamide)dihydrochloride (AIBA) was obtained from Sinopharm Chemical Reagent Co., Ltd. (Shanghai, China). Thiazolyl blue tetrazolium bromide (MTT), Annexin V-FITC Apoptosis Detection Kit, trypsin, Triton x-100, and bovine serum albumin were supplied by Nanjing Jiancheng Bioengineering Institute (Nanjing, China). Folate, N-hydroxysuccinimide (NHS), dicyclohexyl carbodiimide (DCC), dimethylsulfoxide (DMSO), ammonia, ethanol, methanol, and paraformaldehyde were purchased from Sinopharm Holding Chemical Reagent Company, Ltd. (Shanghai, China). SMMC-7721 and H22 cell lines were obtained from the national experimental cell resource platform (Beijing, China). Polyethylenimine (PEI, Mw = 25,000) was obtained from Aladdin Chemical Inc. (Shanghai, China). Other reagents were of analytical/chromatographic grade, and distilled water was used in all experiments.

### 2.2. Synthesis of CQDs

One hundred milligrams PEI was first dissolved in 20 ml deionized water with the concentration of 5 mg/ml. Then, 400 mg citric acid was added to the above solution and stirred for 15 min. Then, the mixed solution was stored in an autoclave and heated for 10 h at 180 °C with the heating rate of 3 °C/min. The resulted solution was centrifuged at 15,000 rpm for the removal of the large particles and the supernatant was purified by dialysis (Mw = 3500) for 6 h (Zhao et al., 2017).

### 2.3. Preparation of HMSN and DOX-HPCF

#### 2.3.1. Preparation of HMSN

First, polystyrene spheres (PSs) were prepared by the emulsification method (Zhang et al., 2009). Then, hollow mesoporous silica nanospheres (HMSN) were prepared by a hard template method. Briefly, 29 ml of distilled water, 12 ml of ethanol, and 1 ml of ammonia were added to a 100 ml conical flask. Then, 0.8 g of CTAB was completely dissolved in the above solution. Ten grams of PSs emulsion was added dropwise to the above mixture over 30 min, followed by the dropwise addition of 4 g of tetraethoxysilane (TEOS). The mixture was centrifuged and washed three times with anhydrous ethanol. The dried product was calcined in air at 550 °C for 5 h.

#### 2.3.2. Preparation of DOX-HPCF

Forty milligrams of DOX was dissolved in 8 ml of pH 7.4 PBS. Then, HMSN (200 mg) was added to the DOX solution, sonicated for 1 h, and stirred for 24 h at 25 °C. The DOX-HMSN was centrifuged.

One hundred milligrams of the above vector was added to 50 ml of Tris buffer (10 mM, pH 8.5). Dopamine (1 mg/ml) was added to the above solution for 3 h at room temperature, and then it was centrifuged. The nanoparticles were referred to as DOX-HP. The DOX-HP was dispersed in 3 ml of Tris buffer, and 2 ml of CQDs (3 mg/ml) was added. The reaction was carried out for 4 h. It was then centrifuged, washed twice with water and lyophilized. The obtained nanoparticles were referred to as DOX-HPC.

DOX-HPC was conjugated with folate active ester. First, 500 mg of FA was added to 10 ml of DMSO containing 0.25 ml of triethylamine. A total of 0.25 mg of DCC and 0.26 mg of NHS were added at room temperature and stirred overnight. The solution was then centrifuged to obtain a supernatant. A mixed solution of acetone/ethyl ether (30% acetone, 70% diethyl ether) was added to the above solution to give a yellow precipitate. It was washed three times with diethyl ether and dried under a vacuum to yield a pale-yellow solid powder, which was a FA active ester. One hundred milligrams of FA active ester was dissolved in 100 ml of water. Then, 100 mg of DOX-HPC (1 mg/ml) was uniformly dispersed in the above solution and stirred for 5 h. It was then centrifuged, washed twice with water and lyophilized. The obtained powder was called DOX-HPCF.

### 2.4. Drug loading of DOX-HPC and DOX-HPCF

Five milligrams DOX-HPC and five milligrams DOX-HPCF were dispersed in 10 ml of deionized water (pH = 4). It was then sonicated for 10 min, left for 1 h, and centrifuged. The collected DOX solution was measured by UV (UV-2000, Unico, Franksville, WI) at a wavelength of 490 nm. The drug loading was calculated according to following formula:

(1) $$\text{LC}\;\left(\text{loading}\;\text{content}\right)\;=\;\frac{\left(\text{weight}\;\text{of}\;\text{loaded}\;\text{drug}\right)}{\left(\text{total}\;\text{weight}\;\text{of}\;\text{nanocomposites}\right)}\times 100\%$$

### 2.5. Characterization of HMSN and HPCF

The morphology and structure of HMSN were observed by TEM (Tecnai G2F30, FEI, Fremont, CA). The zeta potential and particle size of the prepared HMSN, HP, HPC, and HPCF were determined with a laser dispersion particle size analyzer (Nano-ZS90, Malvern, UK). Thermogravimetric analysis (TGA) was performed using a TGA-50 instrument (Shimadzu, Kyoto, Japan). Fourier transform infrared spectroscopy (FTIR) spectra were recorded by an FT-IR spectrometer (IR Affinity-1, Shimadzu, Kyoto, Japan) in the range of 400–4000 cm^−1^. Fluorescence spectra were measured on a Hitachi F-4600 fluorescence spectrophotometer (Hitachi F-4600, Tokyo, Japan). The UV absorption spectra were recorded using a UV (UV-2000, Unico, Franksville, WI) at 490 nm.

### 2.6. Photothermal effect measurement

The photothermal effects of the carriers were measured using an NIR laser (808 nm, Changchun New Industry Optoelectronic Technology Co., Ltd., Changchun, China). Different concentrations of HPCF (50, 125, 250, 500, and 1000 μg/ml) were subjected to 2.0 W/cm^2^ NIR irradiation for 5 min. At the same time, HPCF samples at a concentration of 250 μg/ml were irradiated with different laser powers (0.5, 1, 1.5, and 2.0 W/cm^2^) for 5 min. To determine the photothermal effect of the carrier, the change in the solution temperature was monitored by a thermocoupled thermometer.

### 2.7. *In vitro* drug release study

*In vitro* drug release studies were performed in shakers. The drug release medium was phosphate-buffered saline (PBS, pH = 4 and pH = 7.4). The temperature was 37 °C. First, the loaded sample was added to 20 ml of dissolution medium. Samples (1 ml) were then taken at predetermined intervals and replaced with an equal volume of fresh buffer for further release. Finally, the concentration of DOX was measured by UV (UV-2000, Unico, Franksville, WI) at a wavelength of 490 nm. For the NIR laser irradiation group, the sample was exposed to a laser (808 nm, 2.0 W/cm^2^) for 10 min. Drug release was determined using the methods described above. All experiments were repeated in triplicate.

### 2.8. *In vitro* cell assay

#### 2.8.1. Cell line propagation

The SMMC-7721 cell line was cultured using DMEM containing 10% fetal bovine serum, 1% penicillin, and 1% streptomycin. Fresh medium was replaced every 2 d, and cell digestion was performed using 0.25% trypsin during cell passage.

#### 2.8.2. In vitro cytotoxicity assay

To evaluate the cytotoxicity of DOX-HPC and DOX-HPCF to SMMC-7721 cells, an MTT assay was performed. SMMC-7721 cells were seeded into 96-well plates at 5000 cells/well. After 24 h of culture, pure DOX, DOX-HPC, DOX-HPCF, DOX-HPC-assisted NIR laser irradiation (DOX-HPC + NIR), and DOX-HPCF-assisted NIR laser irradiation (DOX-HPCF + NIR) were suspended respectively in the medium and diluted to various concentrations (the equivalent DOX concentrations were 1000, 500, 250, 125, and 50 ng/ml). For the NIR laser irradiation group, cells were irradiated with an NIR laser (808 nm, 2.0 W/cm^2^) for 10 min. Then, 100 μl of the diluted suspension sample was added to a 96-well plate, and the cells were exposed to the suspension at 37 °C for 48 h. To determine the biosafety of HMSN and HPCF, they were suspended in culture medium. Different concentrations (500, 100, 50, 10, and 5 μg/ml) of the suspension were added to 96-well plates (Qiu et al., 2017). The cells were cultured for 48 h. Then, 20 μl/well of MTT (5 mg/ml) solution was added to a 96-well plate, and cells were cultured for another 4 h in the dark. Next, the supernatant was discarded, and each well was replaced with 150 μl of dimethyl sulfoxide (DMSO) to dissolve the purple crystals. After shaking the sample for approximately 10 min in the dark, the absorbance intensity at 492 nm was recorded on a microplate reader (VERSA max, Molecular Devices, Sunnyvale, CA). Cell viability was calculated using the following formula:

(2) $$\text{Cell}\;\text{viability}\;=\frac{\text{ODt}}{\text{ODc}}\times 100\%$$

ODt represents the absorbance of the treated cells, and ODc represents the absorbance of the control cells.

#### 2.8.3. Cell uptake assay

SMMC-7721 cells were seeded in plates and incubated for 24 h until reaching 80% confluence. Then, the cells were incubated with HPC and HPCF at 50 μg/ml dispersed in DMEM at 37 °C for 1 h. Subsequently, the medium was removed and washed three times with PBS. The cells were fixed with 4% paraformaldehyde solution and 1 μg/ml Hoechst 33342 for 20 min. Next, the cells were immersed in 1 ml of PBS and examined by confocal laser scanning microscopy (CLSM).

#### 2.8.4. Cell apoptosis assay

SMMC-7721 cells were seeded in six-well plates and cultured for 24 h. Pure DOX, DOX-HPC, DOX-HPCF, DOX-HPC + NIR, and DOX-HPCF + NIR (suspended in DMEM, equivalent to 400 ng/ml DOX and 800 ng/ml DOX) were added to the cells and cultured for 48 h. Then, they were digested with trypsin. The collected cells were centrifuged at 1000 rpm for 5 min and washed with PBS, and then centrifuged again. The collected cells were suspended in 500 μl of binding buffer. Then, 5 μl of annexin V-FITC and 5 μl of PI were added to the above suspension.

### 2.9. *In vivo* experiments

Kunming mice (8 weeks, body weight 18–22 g, female) were purchased from the Department of Laboratory Animal Science of Jinzhou Medical University for research. The animal experiment protocol was approved by the Laboratory Animal Ethics Committee of Jinzhou Medical University.

#### 2.9.1. Establishment of ascitic tumors

The H22 liver cancer cell line was cultured in DMEM containing 10% fetal bovine serum, 1% penicillin, and 1% streptomycin. Then, H22 cells (1 × 10^5^) were intraperitoneally injected into Kunming mice. After seven days, swelling of the abdomen was obvious. The ascites was taken out and diluted with physiological saline. Unilateral armpits of the mice were inoculated subcutaneously with 1 × 10^5^ H22 cells. One week later, a distinct tumor was seen in the mice.

#### 2.9.2. Antitumor activity of HPC and HPCF

When the primary tumor volume was approximately 400 mm^3^, 36 mice were randomly divided into six groups: untreated control group, DOX group, DOX-HPC and DOX-HPCF group, DOX-HPC + NIR and DOX-HPCF + NIR group. Among them, four groups of mice were injected with normal saline, pure DOX (5 mg/kg), DOX-HPC, and DOX-HPCF. The remaining two groups were treated with DOX-HPC + NIR and DOX-HPCF + NIR (the tumors were irradiated with an 808-nm laser (2.0 W/cm^2^) for 10 min, 4 h after injection). The drug was given every 3 d, and administration continued for 18 d. Before each dose, the longest diameter and the shortest diameter of the tumor were measured using a Vernier caliper. After the final administration, the mice were euthanized, and the weight of the tumor was measured. The tumor volume was calculated as follows:

(3) $$\text{Volume of tumor}=\frac{{(\text{longest}\;\text{diameter})\times (\text{shortest}\;\text{diameter})}^{2}}{2}$$

The tumor inhibition rate was calculated as follows:

(4) $$\text{Tumor}\;\;\text{inhibition}\;\;\text{rate}=(1-\frac{\text{Wt}}{\text{Wc}})\times \;100\%$$

where Wt is the mean weight of the tumor for each drug treatment group and Wc is the mean weight of the tumor for the control group.

### 2.10. *In vivo* biodistribution study

To assess the tumor targeting of HPC and HPCF, tumor-bearing mice were injected with HPC (5 mg/kg) and HPCF (5 mg/kg) via tail vein. Mice were sacrificed humanely at 3 h after treatment organs (i.e. heart, liver, spleen, lung, and kidney) and tumors were collected immediately. The distribution of HPC and HPCF can be directly tracked by *in vitro* fluorescence imaging of the organs and tumors. The fluorescence intensities were measured with the Bruker In-Vivo FX PRO (In-Vivo FX PRO, Billerica, MA).

### 2.11. Data processing and statistical analysis

All experimental results were expressed as the mean ± SD. The difference between the groups was statistically significant (*p*<0.05).

## 3. Results and discussion

### 3.1. Synthesis and characterization of HPCF and characterization of CQDs

**Figure 1.**
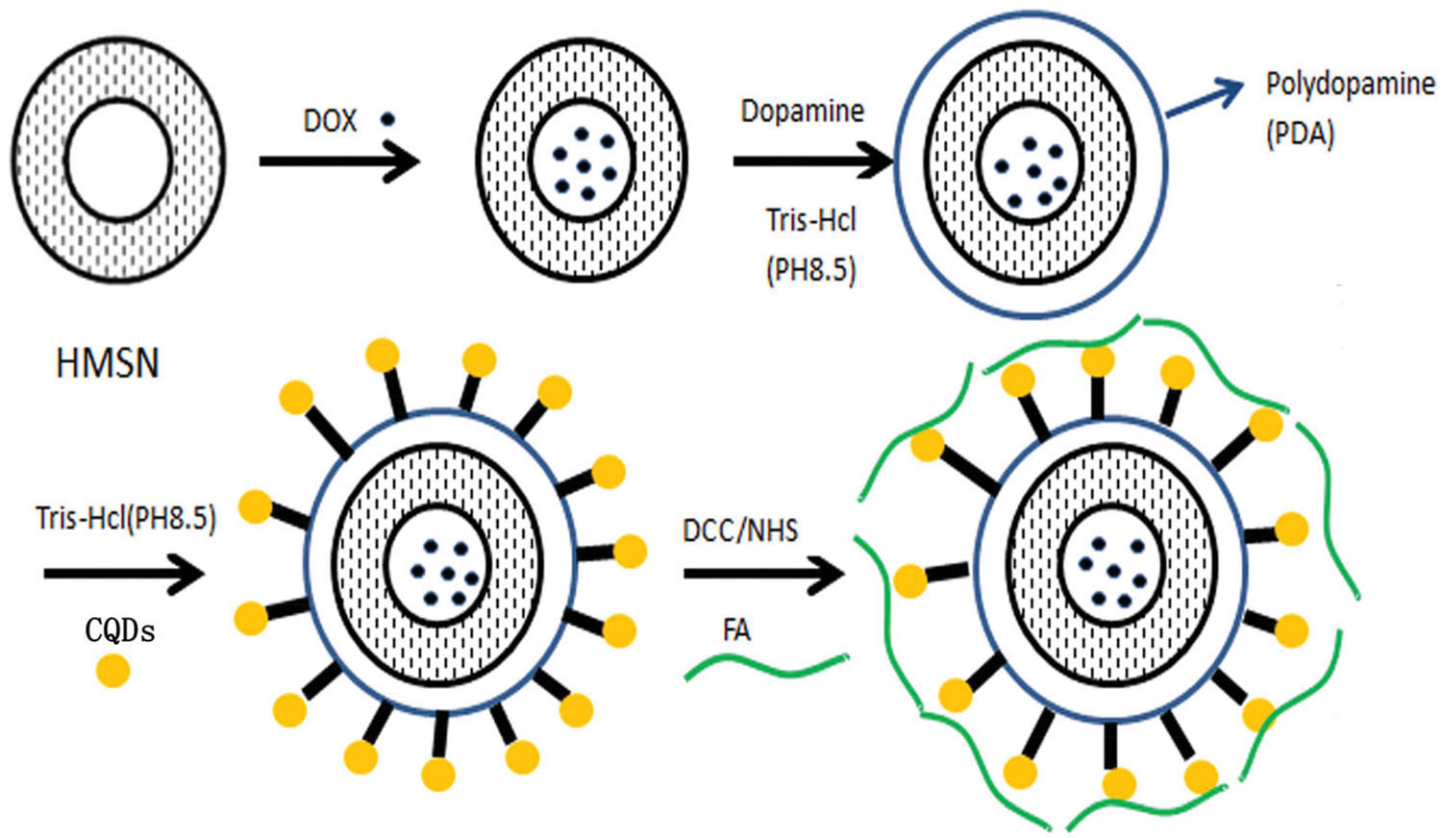
Synthesis scheme for HPCF.

**Figure 2.**
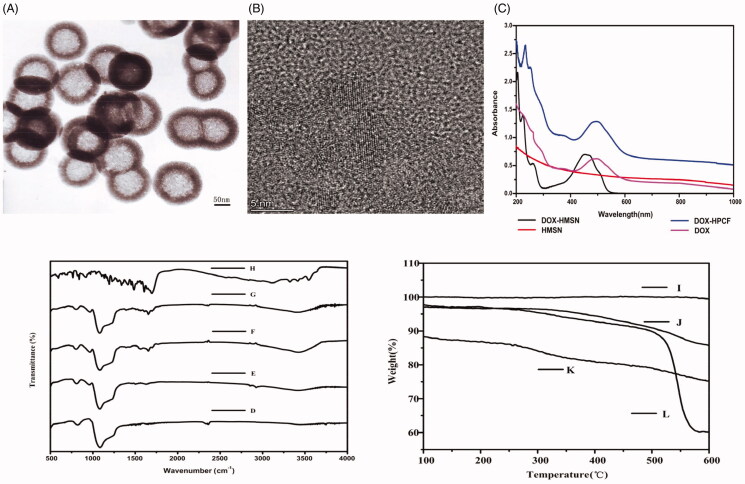
The TEM image of HMSN (A); The TEM image of CQDs (B); UV absorption spectrum of DOX, HMSN, DOX-HMSN, and DOX-HPCF aqueous dispersion (C). The FTIR spectrum of HMSN (D); HP (E); HPC (F); HPCF (G); FA (H); TGA curves of HMSN (I); HP (J); HPC (K); HPCF (L).

The synthesis of HPCF is shown in Figure 1. DOX was loaded into the HMSN by adsorption in PBS at pH 7.4. On this basis, dopamine oxidation self-polymerization formed a PDA coating on the surface of HMSN. The obtained DOX-HP reacted with CQDs according to the Michael addition or the Schiff base reaction (Lee et al., 2007, 2009). PDA and CQDs allowed the delivery system to achieve NIR absorption and biomarker functions (Liu et al., 2012). Finally, FA was coupled with the amino group of CQDs by an amide bond, which enabled the DOX-HPC to achieve targeting due to overexpression of FRs on liver cancer cells. The prepared HMSN was characterized by TEM in order to observe the morphology and structure. As shown in Figure 2(A), the TEM image showed that the HMSN was a monodisperse spherical particle with a hollow structure, and the average size was approximately 140 nm. The mesoporous walls provided a high specific surface area, and the hollow structure was suitable as a drug reservoir. These were the advantages of HMSN as a drug carrier. CQDs were prepared by hydrothermal reaction. Among them, PEI was used as a carbon source. As shown in Figure 2(B), the diameter of the obtained CQDs nanoparticles was about 5 nm. At the same time, in Figure 2(C), we observed the characteristic absorption peak of DOX at approximately 490 nm in the DOX-HMSN and DOX-HPCF spectrum. This indicated DOX was successful loaded into carriers. More importantly, DOX-HPCF showed stronger absorbance in the NIR region than DOX-HMSN. This showed that HPCF had the potential to achieve PTT light conversion.

### 3.2. FTIR characterization of HPCF

In the HPCF preparation process, FTIR was used to characterize whether each reaction step was successful. The results are shown in Figure 2(D). Compared with that of HMSN, the spectrogram of HP showed a characteristic peak at 1560 cm^−1^, which was attributed to the N–H bending vibration of PDA. The characteristic peak at 1650 cm^−1^ was attributed to the tensile vibration of the aromatic ring skeleton of PDA (Zeng et al., 2017). The broad characteristic peak at 3437 cm^−1^ was due to the tensile vibration of NH/OH on the PDA coating. These results proved that PDA was successfully coated on the surface of HMSN. The spectrogram of the graft CQDs on PDA coating showed that the C = N bond was formed according to the characteristic peak at 1660 cm^−1^, indicating that CQDs successfully combined with HP. Finally, the characteristic peaks at 1639 cm^−1^ and 3510 cm^−1^ for HPCF were the tensile vibration of C=O and N–H, respectively. This represented the formation of the amide bond, which proved that FA and HPC were successfully combined.

### 3.3. TGA

TGA was performed to determine the graft amount. As shown in Figure 2(E), compared with the HMSN curve, the weight loss of the HP was 12.9 wt%, indicating that the PDA was successfully coated. The HPC curve compared with that of HP showed a weight loss of 23.4 wt%. This indicated that the graft amount of CQDs was 10.4 wt% after deducting the PDA. In the HPCF curve, the weight loss was 38.9 wt%. After deducting PDA and CQDs, the graft amount of FA was 15.5 wt%. All of these results demonstrated that HPCF was successfully prepared.

### 3.4. Carrier particle size and ZETA potential

During the preparation of HPCF, the particle size and ZETA potential of the sample were characterized by a laser particle sizer. As shown in Table 1, the average size of HMSN was 140.0 ± 6.0 nm, while the average size of HP was 172.5 ± 8.7 nm. Compared with HMSN, the particle size of HP increased approximately 30 nm, which proved that PDA was successfully coated on HMSN. The average size of HPC was 177.2 ± 3.2 nm, and the average size of HPCF was 187.1 ± 11.3. This result indicated that the binding of CQDs and FA only led to a tiny increase in particle size.

**Table 1.** Carrier particle size and ZETA potential of HMSN, HP, HPC, and HPCF.

Polymer	Size (nm)	ZP (mV)
HMSN	140.0 ± 6.0	–36.9 ± 0.6
HP	172.5 ± 8.7	–49.5 ± 0.4
HPC	177.2 ± 3.2	–27.4 ± 0.7
HPCF	187.1 ± 11.3	–38.8 ± 0.3

Zeta potential values are also shown in Table 1. The zeta potential of HMSN was –36.9 ± 0.6 mV, and its negative zeta potential could be attributed to the presence of a large amount of silanol groups on the surface of the silica. After surface modification with PDA, the value dropped to –49.5 ± 0.4 mV. This change was due to the deprotonation of the PDA phenolic hydroxyl group. Since large amounts of amino groups were present on the surface of CQDs, the ZETA potential became –27.4 ± 0.7 after grafting CQDs onto the surface of HMSN-PDA. After HPC was modified with folate, the ZETA potential became –38.8 ± 0.3, which was the contribution of the residual carboxyl group.

### 3.5. UV fluorescence spectrophotometer

**Figure 3.**
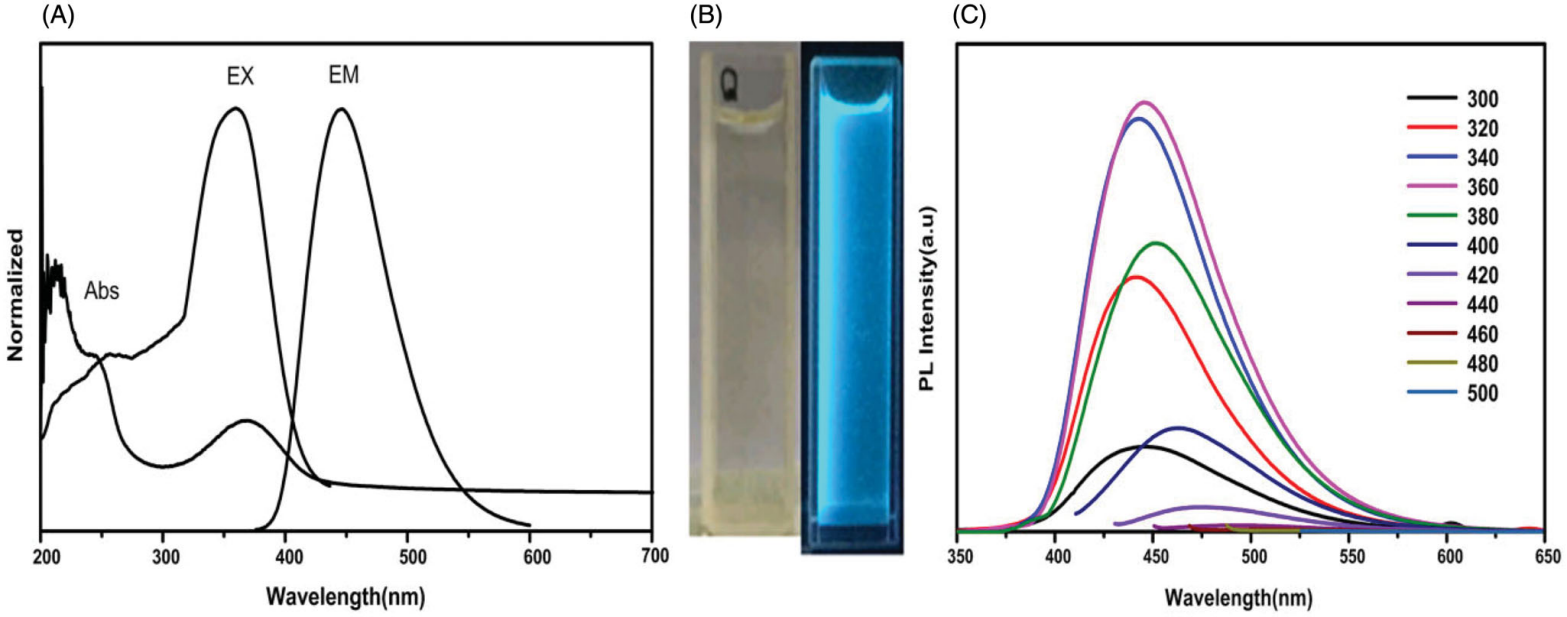
UV–visible absorption spectrum, PL excitation, and emission spectrum (A) of CQDs; The bright field and the bright blue color under 365-nm UV irradiation of the prepared CQDs (B); Photoluminescence (PL) of CQDs at different excitation wavelengths (C).

Transmission electron micrographs of CQDs showed that their particle size was 5 nm. Its UV–visible absorption spectrum showed an absorption peak at 360 nm in Figure 3(A). The fluorescence spectrum of CQDs had the strongest excitation at 360 nm and the strongest emission at 450 nm. As shown in Figure 3(B), this is the bright field and the bright blue color under 365-nm UV irradiation of the prepared CQDs. In addition, as shown in Figure 3(C), photoluminescence (PL) of CQDs was measured by a fluorescence spectrophotometer (Hitachi F-4600, Tokyo, Japan) to evaluate the fluorescent properties of CQDs at different excitation wavelengths. The results indicated that CQDs was not as excitation-dependent. When the CQDs solution was excited in the wavelength range of 300–420 nm, the maximum emission wavelength was still 460 nm, which proved that CQDs had no multi-fluorescence center (Hu et al., 2014).

### 3.6. Photothermal effect measurement

**Figure 4.**
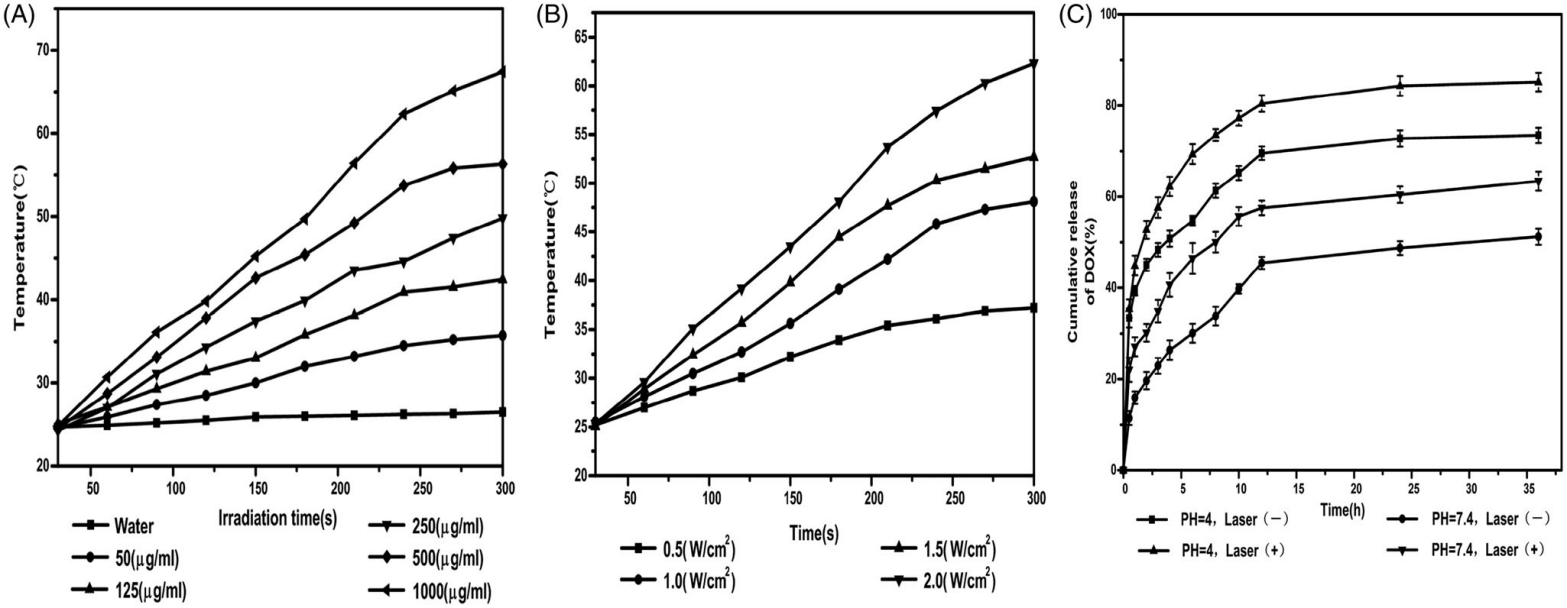
HPCF aqueous dispersions with different concentrations (A); 250 μg/ml HPCF was exposed to laser irradiation with different powers (B); *In vitro* drug release curves of DOX-HPCF at pH = 4, pH = 4 (under NIR laser radiation), pH = 7.4, pH = 7.4 (under NIR laser radiation) (C).

The photothermal effect of HPCF was evaluated due to the strong NIR absorption performance of the PDA. In Figure 4(A), HPCF aqueous dispersions with different concentrations (50, 125, 250, 500, and 1000 μg/ml) were exposed to an NIR laser (808 nm, 2.0 W/cm^2^) for 5 min. The medium temperature ascended with increase in the HPCF concentration. The system temperature of 1000 μg/ml HPCF increased from 24.8 °C to 67.4 °C, while the temperature of H_2_O as the control was only increased by nearly 2 °C. This showed that the photothermal effect of HPCF was concentration-dependent. In Figure 4(B), 250 μg/ml HPCF was exposed to laser irradiation with different powers (0.5, 1, 1.5, and 2 W/cm^2^). The medium temperature under 2 W/cm^2^ NIR irradiation increased from 25.3 °C to 62.3 °C. In contrast, the medium temperature under 0.5 W/cm^2^ NIR irradiation only increased from 25.2 °C to 37.2 °C. Moreover, the medium temperature went up with the increase in irradiation power. This showed that the medium temperature rise of HPCF depended on the NIR laser power level. The above results demonstrated that the HPCF could efficiently convert NIR light into heat and was very suitable as an excellent photothermal agent.

### 3.7. Drug loading and *in vitro* drug release studies

Through UV (UV-2000, Unico, Franksville, WI) measurement, the drug loadings of DOX-HPC and DOX-HPCF were 21.6%±1.6% and 20.6 ± 2.1%, respectively. This indicated that the specific surface area of the modified carrier was slightly reduced, resulting in a corresponding decrease in drug loading. *In vitro* release experiments were used to characterize drug release behavior. In Figure 4(C), the release of DOX from DOX-HPCF reached 51.2 ± 1.8% within 24 h at pH 7.4. Under the same pH conditions, the amount of DOX released reached 63.4 ± 2.1% after laser irradiation. At pH 4.0, the release rate of DOX from DOX-HPCF was higher, and the release amount reached 73.5 ± 1.7% within 24 h. After NIR laser irradiation, the release amount of DOX reached 85.2 ± 2.0% at pH 4.0, which was higher than that under the same conditions without NIR laser irradiation. These results showed that HPCF under acid conditions and laser irradiation could trigger faster drug release. This was primarily due to the heat generated by HPCF under NIR laser radiation, which could disrupt the interaction between DOX and HMSN, thereby inducing DOX release.

### 3.8. *In vitro* cytotoxicity study

**Figure 5.**
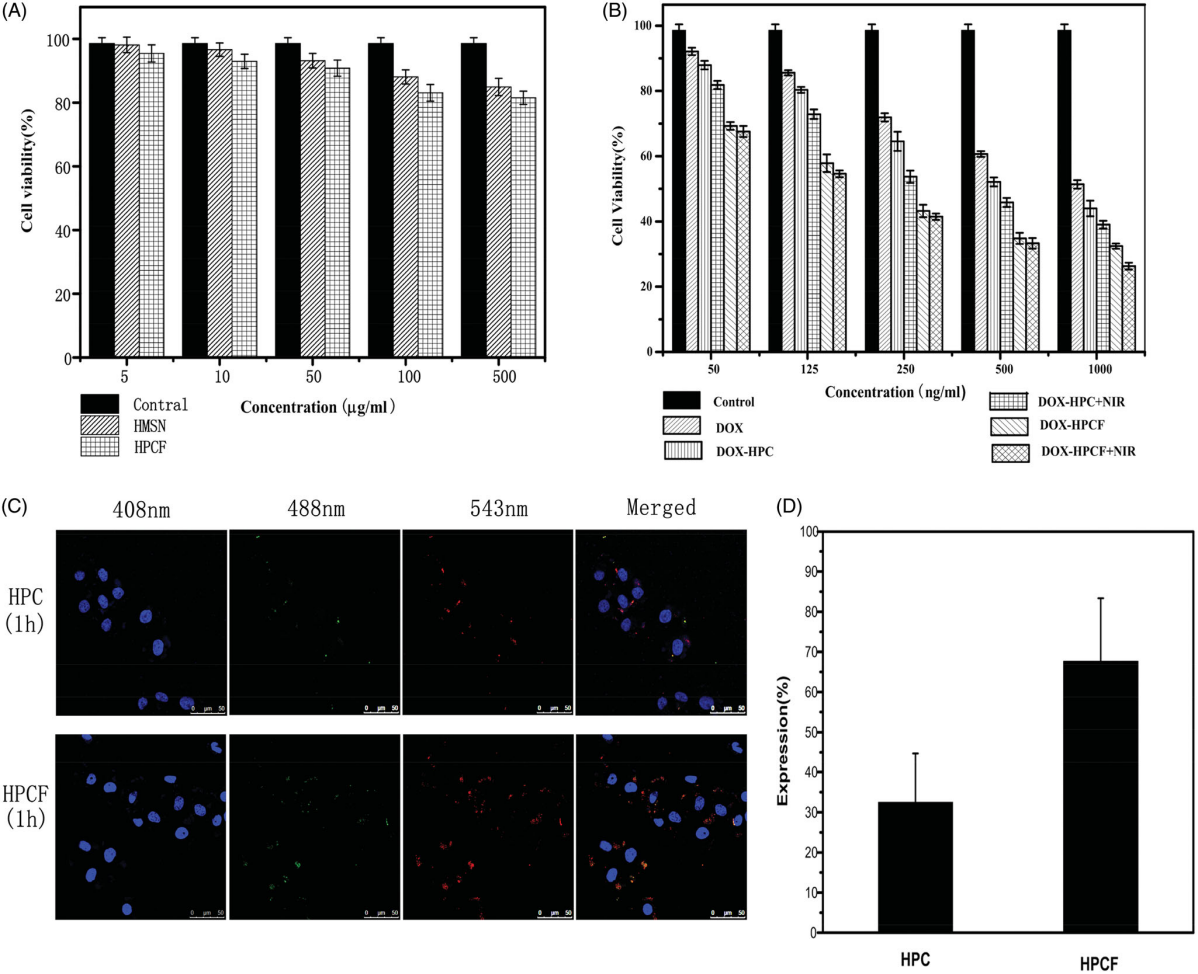
The cell viability of SMMC-7721 cells incubated with HMSN, HPCF (A). The cell viability of SMMC-7721 cells incubated with DOX, DOX-HPC, DOX-HPC + NIR, DOX-HPCF, and DOX-HPCF + NIR for 48 h (B). Data represented as mean ± SD (*n* = 6). Confocal laser scanning microscopy (CLSM) images of SMMC-7721 cells treated with HPC and HPCF for 1 h, respectively (C). The fluorescence intensity of SMMC-7721 cells treated with HPC and HPCF (D).

The cytotoxicity of HMSN and HPCF was evaluated by a MTT assay. As shown in Figure 5(A), the cell viability was over 90% after 48 h of treatment with HMSN and HPCF. This showed that both HMSN and HPCF are biosafe. In Figure 5(B), the cell viabilities of DOX-HPC and DOX-HPCF were significantly reduced compared with that of pure DOX. At a DOX concentration of 250 ng/ml, the cell viabilities of DOX-HPC and DOX-HPCF were 64.5 ± 2.9% and 43.2 ± 1.9%, respectively. The cell viability of pure DOX was 71.9 ± 1.3%. By comparison, the cell viabilities of DOX-HPC and DOX-HPCF-assisted NIR laser irradiation were 53.7 ± 1.9% and 41.4 ± 1.0%, respectively. As the concentration increased, the cytotoxicity of DOX-HPC and DOX-HPCF increased gradually. When the concentration reached 1000 ng/ml, the cell viabilities of DOX-HPC and DOX-HPCF were 44.0 ± 2.4% and 32.5 ± 0.8%, respectively. The cell viability of pure DOX was 51.4 ± 1.3%, and the cell viabilities of DOX-HPC and DOX-HPCF-assisted NIR laser irradiation were 39.0 ± 1.2 and 26.3 ± 1.0%, respectively. The results indicated that DOX-HPCF showed a better cell proliferation inhibition, and DOX-HPCF-assisted NIR laser radiation had the strongest inhibitory effect. This is also illustrated by the calculated half maximal inhibitory concentration (IC50). The IC50 values of DOX-HPC and DOX-HPCF were 648.7 ± 84.1 ng/ml and 264.0 ± 25.8 ng/ml, respectively, while the IC50 of pure DOX was 1101.5 ± 64.8 ng/ml. The IC50 values of DOX-HPC and DOX-HPCF-assisted NIR laser radiation were 416.4 ± 29.5% and 164.5 ± 8.6%, respectively. Obviously, DOX-HPCF-assisted NIR laser radiation showed a better anti-tumor effect. This was the synergistic result of FA targeting and photothermal action.

### 3.9. Cellular uptake assay

The cell uptake of HPC and HPCF was observed by confocal microscopy, as shown in Figure 5(C). At 1 h, the green fluorescence of HPC was weak, indicating HPC had lower cellular uptake. By comparison, the green fluorescence of HPCF was stronger at the same time. This indicated that HPCF had a better cell intake effect. In Figure 5(D), the fluorescence intensities of HPC and HPCF were further analyzed by image J. The results showed that HPCF had higher fluorescence intensity than HPC. This was consistent with the results of ingestion.

### 3.10. Apoptosis detection

**Figure 6.**
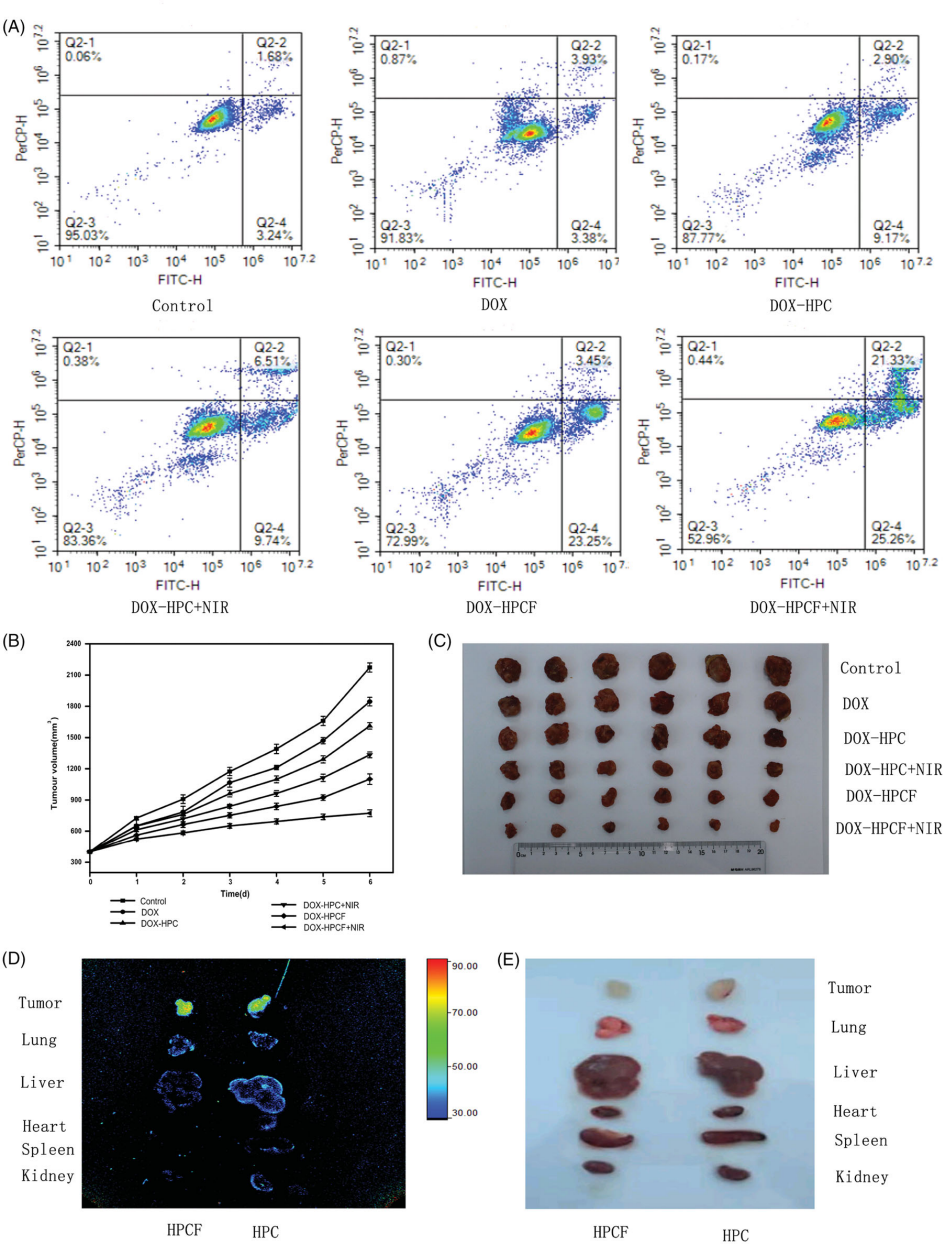
The flow cytometry plots of SMMC-7721 cells treated with control group, DOX group, DOX-HPC group, DOX-HPC + NIR group, DOX-HPCF group, and DOX-HPCF + NIR group(A); The tumor volume of the control group, DOX group, DOX-HPC group, DOX-HPC + NIR group, DOX-HPCF group, and DOX-HPCF + NIR group (B); Data represented as mean ± SD (*n* = 3). Images of tumors from each treatment group following excision on day 21 (C); The fluorescence images of tumor and major organs of tumor-bearing mice from top to bottom: tumor, lung, liver, heart, spleen, and kidney (D); The tumor and organs images of tumor-bearing mice from top to bottom: tumor, lung, liver, heart, spleen, and kidney (E).

Apoptotic cells were detected using flow cytometry. As shown in Figure 6(A), the apoptotic rate of SMMC-7721 cells treated with HPCF was 26.7 ± 3.3%. This was significantly higher than 7.3 ± 1.7% for pure DOX and 12.07 ± 2.6% for DOX-HPC. Due to targeting, SMMC-7721 cells had higher uptake of DOX-HPCF by the endocytic pathway. This led to a high concentration of DOX in the cells, which made it more apoptotic. For the NIR laser irradiation group, the apoptosis rate of SMMC-7721 cells treated with DOX-HPCF-assisted laser irradiation was 46.6 ± 2.8%, which was much higher than that of unilluminated cells. The photothermal therapeutic agent could convert the laser irradiation energy into heat, which caused the carrier temperature to rise and killed the cells (Chai et al., 2018). This indicated that using DOX-HPCF-assisted NIR laser irradiation as a carrier could improve the antitumor effect of DOX. DOX-HPCF was taken up by the FA receptor by receptor-mediated endocytosis. Thus, more DOX was absorbed and released into the cells to induce cell death.

### 3.11. Antitumor effect *in vivo*

*In vivo* anti-tumor experiments showed that DOX-HPCF + NIR significantly inhibited tumor growth. The anti-tumor effects are shown in Figure 6(B). After the last administration, the average tumor volume of the saline group was 2172.6 ± 43.9 mm^3^. The average tumor volume in the DOX group was 1845.0 ± 41.2 mm^3^. The tumor inhibition rate in the DOX group was 15.1 ± 1.9%. In contrast, the mean tumor volumes in the DOX-HPC and DOX-HPCF groups were 1611.8 ± 30.7 and 1100.0 ± 50.3 mm^3^, respectively. The tumor inhibition rate of DOX-HPC was 26.0 ± 1.3%, while the inhibition rate of DOX-HPCF was 49.4 ± 2.3%. The results showed that FA had a targeting effect, which allowed DOX-HPCF to better inhibit tumor growth. For the NIR laser irradiation group, the average tumor volume of DOX-HPC was 1333.5 ± 27.4 mm^3^, and the average tumor volume of DOX-HPCF was 772.7 ± 32.8 mm^3^. The inhibition rates of HPC and HPCF were 38.6 ± 1.3% and 64.4 ± 1.5%, respectively. This confirms that DOX-HPCF + NIR had a better anti-tumor effect. In short, the combination of chemotherapy and phototherapy shows a more significant therapeutic effect.

### 3.12. *In vivo* biodistribution study

After injected HPC and HPCF for 3 h, the fluorescence signals of the main organs of tumor-bearing mice are shown in Figure 6(D). The order from top to bottom are tumor, lung, liver, heart, spleen, and kidney. The fluorescence signal of liver and kidney of HPC was significantly higher than that of HPCF, and the accumulation of HPCF in tumor was more obvious than HPC. The results showed that FA had a good targeting effect and could make HPCF accumulate more in tumors.

## 4. Conclusions

In this study, we successfully prepared DOX-HPCF as a drug carrier for the treatment of liver cancer. *In vitro* drug release experiments showed that DOX-HPCF was pH-sensitive. Cell uptake experiments showed that HPCF increased cell uptake and showed good targeting to SMMC-7721 cells. *In vitro* cell experiments and *in vivo* tumor-bearing experiments showed that the drug delivery system combined with PTT could effectively inhibit the growth of liver cancer cells. Together, these results indicated that HPCF could effectively deliver DOX to liver cancer cells and synergistically promoted apoptosis under photothermal action. Therefore, HPCF is a promising vector for the therapy of hepatocellular carcinoma.
